# Spatiotemporal patterns in British racing and equestrian sports: Implications for pathogen transmission

**DOI:** 10.1111/evj.70126

**Published:** 2025-12-05

**Authors:** Tegan A. McGilvray, Kim B. Stevens, Kelsey L. Spence, Sarah M. Rosanowski, Josh Slater, Jacqueline M. Cardwell

**Affiliations:** ^1^ Royal Veterinary College London UK; ^2^ University of Guelph Ontario Canada; ^3^ Equine Veterinary Consultants (EVC) Limited Hong Kong Hong Kong; ^4^ Department of Veterinary Clinical Sciences, Faculty of Veterinary and Agricultural Sciences University of Melbourne Werribee Victoria Australia

**Keywords:** equestrian event, horse, infectious disease, racehorse, spatial, sport

## Abstract

**Background:**

The widespread assumption that there is minimal potential for pathogen transmission between British racehorse and sport horse populations remains unverified by empirical evidence.

**Objectives:**

To characterise spatiotemporal patterns of horse attendance at racing and other sport events in Great Britain in 2018.

**Study Design:**

Spatiotemporal analysis.

**Methods:**

Publicly available data from British Horseracing Authority, British Dressage, British Eventing, Endurance GB, and British Showjumping events in Great Britain during 2018 were analysed. Horse attendance was summarised by discipline, month, and season. Venue density was mapped with kernel density estimation. Sport venues within 5 km of racecourses with horse attendance within 24 h of racing were identified and Kulldorff's spatial scan statistic was used to detect significant time–space clustering of venue use.

**Results:**

Excluding showjumpers, 49,012 horses competed in 8314 events across 598 venues during 2018, generating over 400,000 horse–venue attendances. Most horses (97.2%; *n* = 47,635/49,012) competed in a single discipline. Venue attendances peaked in summer and were concentrated in southeast England. There were five significant space–time clusters of venue‐events within 5 km and 24 h of each other involving 5 racecourses and 8 sport venues. The most likely cluster was in the southeast of England, between January and July, with a relative risk of 62.54.

**Main Limitations:**

Inconsistent horse identification precluded horse‐level analysis of showjumping data.

**Conclusions:**

Racehorse and sport horse populations competing in Great Britain are largely separate, but limited opportunities for local or indirect pathogen spread do exist during peak seasons in areas with high venue density.

## 1 INTRODUCTION

In Great Britain, Thoroughbred (TB) racing and nonracing sport horse populations are widely regarded as separate in terms of infectious disease risk, management, and prevention. The TB racing industry is subject to strict regulations and biosecurity protocols enforced by the British Horseracing Authority (BHA),^1^, ^2^, ^3^ and is also guided by the Horserace Betting Levy Board (HBLB) International Codes of Practice.^4^ The nonracing sector does not have the same centralised regulatory oversight, nor a single governing body to enforce uniform requirements across disciplines. Instead, rules and recommendations vary between affiliated bodies and jurisdictions, often relying on individual owners' and managers' practices, creating challenges for both understanding and enforcement of biosecurity.^5^, ^6^ While affiliated equestrian disciplines such as British Dressage, British Eventing, Endurance GB, and British Showjumping have some biosecurity guidelines and vaccination requirements in place,^7^, ^8^, ^9^, ^10^, ^11^ variation in implementation is likely, both within and across disciplines. Any connectivity between sectors raises concerns about the potential for pathogen transmission, particularly for the more tightly regulated racing and elite sport horse populations. However, it is also relevant for populations competing at lower levels where shifting vector distributions and contact with more widely travelled horses could pose a risk of exposure to exotic or emerging pathogens. These risks are especially challenging, as pathogens can spread between individual horses through direct or indirect contact, locally via airborne or vector transmission, and between populations through infected horses' movements.^6^, ^12^, ^13^, ^14^, ^15^, ^16^, ^17^, ^18^, ^19^ Given the growth of the wider equestrian industry, with the economic value having recovered to pre‐COVID‐19 pandemic levels,^20^ and the rising threat of vector‐borne disease outbreaks due to climate change,^12^, ^13^, ^19^ an up‐to‐date understanding of the spatiotemporal distribution and connectivity of British performance horses is crucial for improved disease prevention and control strategies.^12^, ^13^

The perception that racehorses are effectively not at risk from pathogens circulating within or between other sport horse populations assumes spread through direct contact only, overlooking the potential for indirect local spread. Although this is relatively uncommon, such spread has been documented, for example in the 2007 equine influenza outbreak in Australia.^14^ Geographical areas with high densities of venues and movements are likely to be highly connected through horse movements, shared personnel or equipment, overlapping event dates, and short‐range airborne spread,^15^, ^16^, ^21^ all of which can facilitate pathogen transmission. Furthermore, the location of areas with high densities of venues and frequent horse attendance is likely to vary throughout the year, because of seasonal patterns in both racing and other equestrian competitions,^22^, ^23^ leading to corresponding fluctuations in the potential for pathogen transmission between disciplines.

Studies conducted over 10 years ago on the spatial distribution of sport and leisure horses in Great Britain^17^, ^18^, ^19^ identified significant geographical variations in horse density, with the highest densities in England, followed by Wales and Scotland, as well as substantial movement of horses between equestrian venues.^20^ Previous research has also highlighted the internationally interconnected nature of the equine industry.^16^, ^24^, ^25^, ^26^ Furthermore, spikes in connectivity, caused by attendance at competitions, sales and other gatherings, can lead to locations acting as ‘hubs’ facilitating pathogen transmission. Sudden increases in the number of horses in contact at these events can connect otherwise separate groups, and network analyses have highlighted the potential for horses to act as bridges between locations and for clustering of horses within sub‐networks.^22^

However, since the research by Boden et al.,^17^ Robin et al.^18^ and Robin et al.^19^ was conducted, there has been substantial growth in the British equine industry, with increased numbers of equestrian facilities, and participation in equestrian sport.^20^, ^27^ An updated assessment of horse distribution and movements is required to allow for evidence‐based disease preparedness planning. Understanding the structure of the equine industry, comprising racing, sport (nonracing), leisure and working horses, and how these sectors are organised, regulated and interact through horse movements, venue use and management practices, is essential for designing effective biosecurity measures, targeted surveillance and disease control strategies. Precise identification of high‐risk areas and the potential for pathogen spread between performance horse populations enables focused prevention efforts and supports the development of targeted interventions such as zoning and compartmentalisation.^28^

The aim of this study was therefore to explore the spatiotemporal characteristics of performance horse events in Great Britain. Specific objectives were to (i) describe the geographical distribution of racecourses and other equestrian venues, (ii) estimate annual numbers and seasonal patterns of horse‐venue attendance at these venues and (iii) identify spatiotemporal clusters of events that create the potential for pathogen transmission between racehorses and other sport horses.

## 2 METHODS

### 2.1 Data sources, extraction, cleaning and manipulation

This study examined publicly available data from BHA fixtures and British dressage, endurance, eventing, and showjumping events that took place in Great Britain in 2018, which at the time of analysis was the most recent year in which racing and equestrian sporting calendars had not been disrupted by the COVID‐19 pandemic.

Racing and showjumping data were obtained directly from the BHA and British Showjumping, on request. These data included horse identification (number or name), race or competition date, and racecourse or competition venue name and postcode. An online web‐scraping application (https://webscraper.io) was used to extract publicly available archived attendance data for British Dressage (https://www.britishdressage.co.uk/), Endurance GB (https://www.endurancegb.co.uk/) and British Eventing (https://www.britisheventing.com/), by prior arrangement and in accordance with the respective website terms and conditions. All data were extracted on 01/09/2020; variables obtained were horse name, rider name, competition date, venue name and postcode, or venue address.

All datasets were checked for missing or implausible values and cleaned to ensure consistency across variables. Horse and venue names were standardised to allow accurate matching across events, for example by converting all to title case and correcting obvious spelling errors. Where differences were minor (e.g., missing accents or punctuation), potential duplicates were flagged for manual review, and validated by cross‐checking horse, venue, show, and rider details where possible. Where information was unclear, online profiles on competition results pages or rider databases were searched to confirm whether records referred to the same horse. Although most horses had only one associated rider, in some cases multiple riders were linked to a single horse, limiting the ability to validate these names. Showjumping data presented additional challenges. There were frequent discrepancies in horse name spelling and many potential duplicate names with no reliable way to confirm identities. As a result, showjumping data were only used in venue‐level analysis, and not horse‐level analysis. For dressage, endurance, and eventing, if cross‐checking was not possible, show names that were identical or differed only by the presence or absence of diacritics, were assumed to indicate the same horse, as competition regulations typically require unique horse names within disciplines.

Venues used by a single discipline were classified by that discipline, while venues hosting more than one discipline were classified as multidisciplinary. The main use of each multidisciplinary venue was defined by its most frequently hosted discipline. Any horse that competed in more than one discipline was categorised according to the discipline in which it had most frequently competed. Postcodes that were not initially available were identified by searching for venue websites online and retrieving the postcode listed in the venue's contact or address details. All postcodes were converted to British National Grid coordinates using an online conversion tool (https://gridreferencefinder.com/postcodeBatchConverter/).

The term ‘event’ is used throughout the text to refer to any racing fixture, competition or show. ‘Racecourses’ refers to BHA‐regulated racecourses, ‘sport venues’ refers to locations where nonracing events are held, and ‘venues’ is used as a generic term for both. ‘Racehorse’ refers to a horse competing in BHA racing and ‘sport horse’ refers to horses competing in dressage, eventing, endurance or showjumping. ‘Local pathogen spread’ refers to the transmission of pathogens over short distances via indirect or environmental routes, rather than direct animal movement.

### 2.2 Data analysis

#### 2.2.1 Horse and venue characteristics

The number and proportion of horses competing in each discipline (excluding showjumping), and monthly and seasonal distribution of events, were determined. Seasons were defined as spring (March, April, May), summer (June, July, August), autumn (September, October, November) and winter (December, January, February). Event dates were used to determine the number of events occurring each month and the number of multiday events. Each date of a multiday event was counted as a separate event day. When a horse competed at one venue on consecutive dates, each attended day was counted as an individual data point. The distributions of attendance numbers and distances between venues were checked using the Shapiro–Wilk test in Stata 17 (StataCorp) and summarised using medians, interquartile ranges (IQR) and percentage change, or means and standard deviations (SD), as appropriate.

#### 2.2.2 Spatial analysis

ArcGISv 10.8.1 (Environmental Systems Research Institute) was used for all spatial analyses and manipulations, unless otherwise indicated.

##### Density of venues and distance between venues

Kernel‐smoothed maps of venue density were generated for each of the five disciplines, and all disciplines combined, to identify areas with high densities of discipline‐specific venues, using a bivariate Gaussian kernel density estimation and a bandwidth of 40,000.

##### Identification of clusters of venues at risk of local pathogen transmission between racecourses and sport venues

To identify clusters of venues with potential for local pathogen spread between racecourses and sport venues, sport venues were selected based on two criteria: (i) location within a 5 km radius of a racecourse, and (ii) horse attendance at both the racecourse and the sport venue on the same day or within 24 h (defined as the day before, the day of, or the day after attendance at the racecourse). This was an exploratory analysis, intended to highlight short‐term, localised patterns that could be relevant for the transmission of a range of pathogens, including endemic, exotic and novel. These spatial and temporal parameters were not intended to model a specific pathogen but were based on the epidemiological characteristics of equine influenza as a representative example of a respiratory pathogen that causes outbreaks.^14^, ^29^ The 5 km radius accounts for potential local transmission via indirect routes such as shared personnel, vehicles, or other fomites,^14^, ^29^, ^30^ as well as airborne transmission, which may occur up to 2 km under favourable environmental conditions.^14^ This is consistent with previous studies of short‐range equine movement and pathogen spread,^14^, ^29^, ^30^ although the full extent of network interactions is unknown and may exceed 5 km, especially without data on horses' home premises or travel routes. The 24‐h window used in the main analysis was selected to reflect a range of plausible transmission scenarios, with aerosol spread between venues within a short timeframe considered the most plausible. Using a 24‐h period rather than a strict ‘same day’ cutoff allowed for natural variations in event timing and attendance, recognising that horses may arrive, depart, or overlap at venues at different times throughout the day. This approach accounted for transmission that could occur late on one day and early the next, which a strict same‐day definition might miss. It also reflects the expectation that disinfection and other biosecurity measures likely reduce transmission risk beyond this period. Kulldorff's focal space–time scan statistic (SaTScan 9.6. Harvard Medical School and Harvard Pilgrim Health Care) was used to identify clusters of sport venues located within 5 km of a racecourse and with horse attendance within 24 h of racing (defined as ‘cases’; all other nonracing venues defined as ‘controls’). Racecourses matched with ‘cases’ were used as the foci of the statistic. A Bernoulli probability model was used with spatial and temporal windows both set to 50% of the population at risk to allow detection of clusters across a broad range of cluster sizes, following recommended practice,^31^ and Monte Carlo randomisation with 999 permutations. SaTScan controls for multiple testing implicitly by using Monte Carlo replications of the maximum likelihood ratio under the null hypothesis. The *p*‐value reflects how extreme the observed most likely cluster is, relative to the distribution of the maximum cluster statistic across 999 permutations, rather than testing each candidate cluster separately. The observed‐to‐expected (O/E) case ratio was calculated as the number of observed cases within a cluster divided by the expected number of cases under the null hypothesis of equal risk inside and outside the cluster.^31^ This reflects the estimated risk within the cluster relative to that outside the cluster. Relative risk (RR), defined as the incidence within the cluster compared to that outside the cluster, was also calculated to quantify the strength of detected clusters. SaTScan reports the RR for each detected cluster and a single *p*‐value based on the maximum likelihood ratio, reflecting the statistical significance of the most likely cluster. Unless otherwise stated, RR values describe the magnitude of cluster intensity and are not subject to statistical testing.

## 3 RESULTS

### 3.1 Description of dataset

Excluding showjumpers, 49,012 individual horses participated in 8314 equestrian events (including showjumping) at 598 distinct venues across Great Britain during 2018. There was a total of 404,592 attendances, by a population comprising 39.3% Thoroughbred racehorses (*n* = 19,252/49,012) and 60.7% sport horses (*n* = 29,760/49,012). Events took place on every day of the year apart from Christmas Eve and Christmas Day. Racing took place on 360 out of 365 days (99%), dressage on 350 days (96%), showjumping on 341 days (93%), eventing on 152 days (41.6%), and endurance on 79 days (21.6%). Overall, there were 1471 racing, 3053 dressage, 385 eventing, 195 endurance, and 3420 showjumping event‐days with 132 instances of events spanning two or more days.

### 3.2 Horse‐ and venue‐level multidisciplinarity

All racehorses competed exclusively in racing, at racecourses, and throughout all months of the year. Among the 29,760 sport horses, the vast majority (95.4%; *n* = 28,383) competed in only one discipline. The most common disciplines were eventing (45.4%; *n* = 13,502) and dressage (42.9%; *n* = 12,754), with a smaller proportion participating in endurance (7.0%; *n* = 2087). Only 1417 sport horses, representing 4.8% of the total sport horse population (*N* = 29,760) and 2.9% of all sport and racehorses (*N* = 49,012), competed in multiple disciplines. The most frequent combinations involved dressage and endurance (0.26%; *n* = 77/29,760), eventing and endurance (0.17%; *n* = 51/29,760), and dressage, eventing and endurance (0.03%; *n* = 9/29,760).

Numbers and proportions of venues that hosted each equestrian discipline, or a combination of disciplines, and numbers of events per discipline, are summarised in Table 1. Despite racehorses being the largest subgroup of performance horses (*n* = 19,252), only 10.0% of all venues (*n* = 60/598) were racecourses. Only three of the 598 venues (0.3%), all of which were racecourses, hosted a combination of racehorse and sport horse events.

**TABLE 1:** Number and percent of the 598 British equestrian venues hosting each equestrian discipline, or combination of disciplines, in 2018.

	Venues		Events
	%	*n*	*n*
Single discipline			
Racing	9.5	57	1471
Dressage	15.4	92	3053
Eventing	10.9	65	385
Showjumping	23.6	141	3420
Endurance	20.4	122	195
Multidisciplinary			
Racing and eventing	0.17	1	
Racing and showjumping	0.17	1	
Racing, dressage, eventing, showjumping	0.17	1	
Other (nonracing) combinations	19.7	118	
**Total**	**100**	**598**	

### 3.3 Temporal distribution of discipline‐specific events and horse‐venue attendances

**FIGURE 1:**
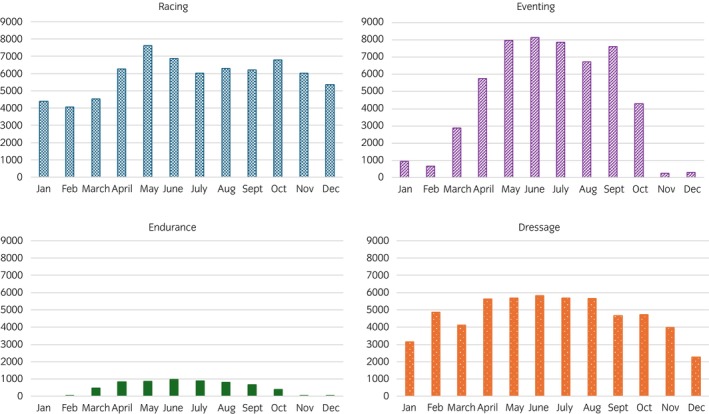
Number of individual British performance horses competing per discipline by month in 2018.

Although racing, dressage and eventing took place throughout the year, all three disciplines showed a degree of seasonality, with increased activity over the spring and summer months. Endurance displayed similar seasonality but was the only discipline that did not compete in January. The overall number of horses racing and competing peaked during the summer (June–August), with 36.3% (*n* = 146,738/404,592) of attendances (counting multiple attendances by the same horse) across all disciplines happening during those months. Discipline‐specific peaks in attendances occurred in August for dressage (11.4%; n = 14,555/127,291), June for endurance (17.6%; *n* = 2052/11,627), and May for both eventing (18.1%; *n* = 31,104/172,234) and racing (11.0%; *n* = 10,286/93,439). In contrast, the number of individual horses racing peaked in May (39.5%; *n* = 7606/19,252), while individual dressage (41.3%; *n* = 5831/14,120), endurance (43.4%; *n* = 966/2224) and eventing (54.8%; *n* = 8139/14,842) horse numbers peaked in June (Figure 1). Median attendances rose markedly from autumn–winter to spring–summer across all disciplines. The largest seasonal increases were observed in endurance and eventing, with median attendances rising from 40 (IQR 573) to 1769 (IQR 391.5, +97.7%) and from 1035.5 (IQR 8322.25) to 24,396.5 (IQR 3808.75, +95.8%), respectively. Dressage and racing showed more moderate seasonal variation, increasing from 7477.5 (IQR 4043.75) to 13,413.5 (IQR 2051.75, +44.3%) and from 6341.5 (IQR 1870.25) to 8804.5 (IQR 928.75, +28.0%), respectively (Figure 1).

### 3.4 Geographic distribution of venues

**FIGURE 2:**
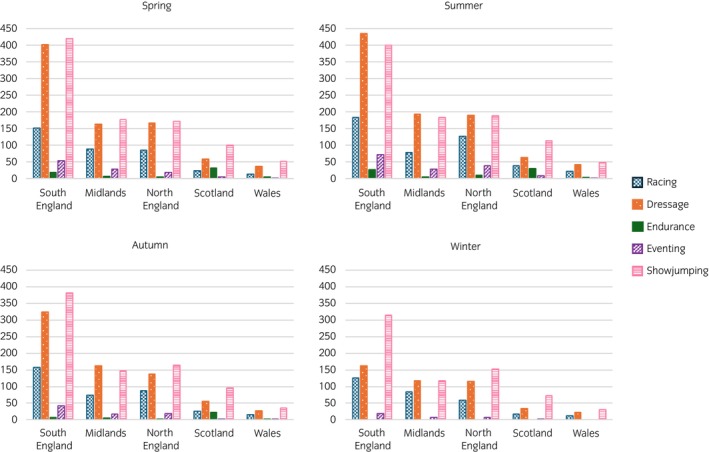
Geographical and seasonal distribution of the number of races and sport horse events in Britain in 2018.

Of the 598 venues, 72.9% (*n* = 436) were in England, 19.6% (*n* = 117) in Scotland and 7.5% (*n* = 45) in Wales. Figure 2 shows the regional distribution of events by discipline across the four seasons. Throughout the year, events for all disciplines were most frequent in the south of England, except for endurance, which predominantly took place in Scotland. The Midlands and northern regions of England had similar event numbers, particularly in spring and summer, for dressage and showjumping. Wales consistently hosted the fewest events across all seasons and disciplines, with Scotland hosting only slightly more.

**FIGURE 3:**
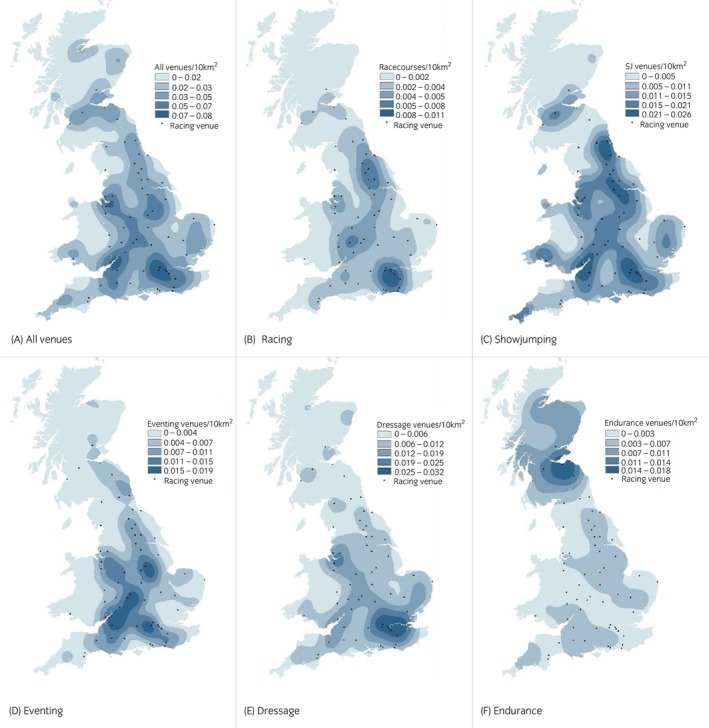
Kernel‐smoothed density maps of (A) all racing and sport venues, (B) British Horseracing Authority racecourses, (C) showjumping venues, (D) eventing venues, (E) dressage venues and (F) endurance venues in Britain in 2018 (1 km^2^ grid cells and a bandwidth of 40,000).

Venues in England were reasonably evenly dispersed; those in Scotland were clustered centrally, and those in Wales were primarily situated along the southern coastline and the England–Wales border (Figure 3A). There was a noticeable concentration of racecourses in the southeast of England, and two further areas with high densities of racecourses in the northeast and the west of England (Figure 3B). Showjumping was the most dispersed discipline, with high densities of venues in numerous areas throughout England, Scotland, and Wales (Figure 3C). Eventing venues were concentrated in southern and central England (Figure 3D), and dressage venues had two distinct high‐density areas in southeast England and northwest England (Figure 3E). Endurance venues were largely geographically separate from the other disciplines, with the highest venue‐density in Scotland (Figure 3F).

### 3.5 Spatiotemporal proximity of racing and nonracing events

**FIGURE 4:**
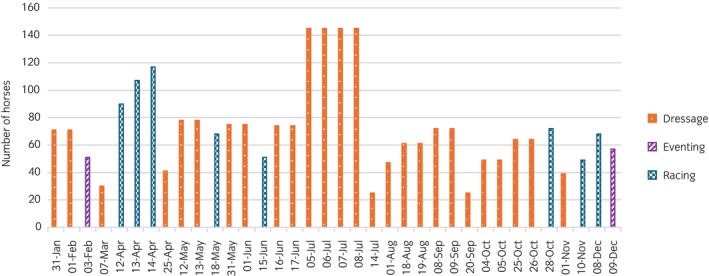
Distribution of sport horse attendance at the single British Horseracing Authority racecourse (Racecourse C) that hosted racing, eventing and dressage events in 2018. This racecourse also hosted showjumping from January to March and August to November, but reliable attendance figures were unavailable. Horses from different disciplines never attended on the same day.

Two of the three racecourses that hosted events other than racing hosted only one other discipline, and only during 1 or 2 months of the year. Racecourse A, located in Wales, hosted eventing on 2 days in the summer, one in June and one in July, with 474 horses attending on each of these days. Racecourse B, located in northwest England, hosted showjumping on 1 day in May (exact horse numbers could not be calculated due to horse name discrepancies). There was only one truly multidisciplinary racecourse, Racecourse C, which hosted dressage (January–November), showjumping (January–March and August–November) and eventing (February and December) in addition to racing. However, horses from different disciplines never attended on the same day. Only two racecourses (A and C) had fixtures where nonracing attendees were present within 3 days prior to racehorse attendance. Racing and sport events never occurred on the same day, and a sport event never took place the day before racing at either venue. On two occasions, a sport event occurred 2 days before racing (Racecourses A and C), and on one occasion a sport event occurred 3 days before racing (Racecourse C) (Figure 4).

Of 18 racecourses located within 5km of 23 sport venues, 11 had been attended within 24 h of a sport event, on 137 occasions (1.6% of the 8314 total fixtures and events). Racehorse and sport horse attendances had occurred on the same day on 45 occasions; a further 45 involved sport horses attending the day before racing, and 47 involved racehorses attending the day before sport events. However, only five of the 11 racecourses and 8 of the 23 sport venues (four of which hosted more than one discipline) formed part of a significant spatiotemporal cluster. Details of the five identified clusters are provided in Table 2. These were located throughout England and occurred at different times of the year, with the most likely cluster in the first half of the year and the remaining four clusters in the second half of the year. Distances between racecourses and nonracing venues ranged from 0 to 5.0 km, with a mean of 2.9 km (SD 1.4 km).

**TABLE 2:** Results of Kulldorff's focal space–time scan analyses in which racecourses comprised the focal points of the analysis around which significant (*p* < 0.001) clusters of sport venues within 5 km of the racecourse and holding an event within 24 h of the racing fixture, in 2018, were identified.

Cluster (<5 km and ≤24 h)	Location of racecourse	Significant period	Radius (km)	Observed/expected cases	Relative risk (RR)	Number and type of sport venue with number of attendees during significant period (excluding SJ numbers)
1	SE England	12/01–01/07	4.6	44.3	62.5	2 showjumping 1 dressage (*n* = 272)
2	West Midlands	06/05–18/10	3.5	15.4	17.4	1 showjumping and dressage (*n* = 561)
3	Yorkshire and the Humber	23/05–31/10	3.0	20.4	22.4	1 showjumping, dressage and eventing (*n* = 2463)
4	Yorkshire and the Humber	06/07–24/07	3.1	59.5	62	1 showjumping, dressage and eventing (*n* = 33)
5	NW England	06/07–16/12	3.8	39.6	41.7	1 showjumping 1 dressage (*n* = 40)

O/E ratios of these clusters ranged from 15.4 to 59.5, indicating that the number of observed sport venues located within 5 km of a racecourse and attended within 24 h of racing (‘cases’) was many times greater than expected under a random distribution. Corresponding RRs ranged from 17.4 to 62.5, indicating markedly elevated risks. Note that RR values are reported as descriptive indicators of relative intensity. Confidence intervals (CIs) are not implemented in standard SaTScan outputs and would require custom post‐processing. Therefore, RR values should be interpreted with caution as point estimates without associated precision. Given the primary objective of detecting high‐risk clusters rather than estimating exact risk magnitudes, RR values were used for relative comparison only. The most likely cluster was in the southeast of England with 44.3 times more sport venues located within 5 km of a racecourse and attended within 24 h of racing (‘cases’) observed than expected, and a RR of 62.5 indicating that sport venues within this cluster were over 60 times more likely to be within 5 km and attended within 24 h of their local racecourse compared to those located outside of the cluster. The racecourse within this cluster was also the most highly attended racecourse located within 5 km of a sport venue, with 4933 racehorse attendances overall during 2018, of which 55.5% (*n* = 2737/4933) occurred during the temporal window of the cluster (12 January – 1 July). The second most likely cluster included a racecourse in the East Midlands that received 3370 racehorse attendances during 2018, of which 22.1% (*n* = 745/3370) were during the temporal window of the cluster (6 May – 18 October).

## 4 DISCUSSION

This is the first study to characterise the proximity of British racehorse and sport horse populations in space and time over the course of a year and provide data‐driven insight into potential routes for pathogen transmission between these groups. Our main finding supports the long‐held belief that the racing and sport horse sectors operate largely separately in terms of spatial and temporal proximity between events, with consequently few opportunities for direct or indirect contact that might facilitate pathogen transmission between the sectors. Notably, no venues were used by both racehorses and sport horses on the same day, indicating that direct contact at competitions is unlikely to be a key route of transmission, although small windows for potential indirect or local transmission were identified. However, our data do not capture horses' home locations or the movements of personnel—factors that may influence how distinct these populations remain outside the competition setting.

Previous work examining static spatial horse distributions in Britain indicated that horse densities were higher in England and Wales than in Scotland, particularly in the east and southeast of England.^17^, ^18^, ^19^ Our spatial mapping highlighted that in the southeast of England, where racecourses were most densely concentrated, there was also a large number of showjumping and dressage venues, and to a lesser extent, eventing venues. Together these findings suggest that the frequent horse attendance at venues in the southeast of England potentially creates conditions conducive to pathogen transmission between racing and other sport horse populations, and across the performance horse population as a whole.

The southeast of England is a key geographical area of concern for vector‐borne pathogens, as competent vectors such as *Culex modestus* (West Nile virus) and *Culicoides* spp. (African horse sickness) are present.^12^, ^32^ The primary concern in such contexts is the overall number of individual horses at risk in areas with high horse attendance, which may facilitate their exposure to local vectors. Similarly, in the northeast and Midlands there was a high geographical concentration of eventing, showjumping, and racing venues. While this region has not historically been a focus for vector‐borne disease, the recent identification of West Nile virus genetic fragments in mosquitoes in Nottinghamshire highlights emerging vector‐related risks in such high‐density equine areas.^33^ Although West Nile virus is not transmitted directly between horses, and horses are dead‐end hosts, its inclusion in this discussion is to highlight individual horse‐level exposure risk, rather than direct transmission between venues or sectors. Such risks remain important for disease prevention and awareness, even though they do not contribute to the formation of transmission clusters in the way that contagious horse‐to‐horse diseases might.

Our analysis of seasonal patterns showed that event attendance peaked in spring and summer across all nonracing disciplines, aligning with the core periods of both the Flat and National Hunt racing seasons. Several major racing fixtures also occur during this time, including the Cheltenham Festival (March), Grand National Festival (April), Guineas Meeting (May), Royal Ascot (June), Glorious Goodwood (late July to early August), St Leger Festival (September), and the Cambridgeshire Meeting (September).

Although only three venues hosted both racing and nonracing events, and never concurrently, there were instances of sport horses attending competitions held at racecourses within 3 days of racing. In all cases, racehorses had attended the venue first. This directionality, if consistent, is likely to affect transmission dynamics, with a greater likelihood of pathogens being introduced to the sport horse population from the racing population than vice versa. Racecourses undergo standardised disinfection following events,^3^ likely reducing residual pathogen viability. The physical layouts at these sites are also designed to separate different groups,^2^ helping to reduce pathogen transmission. Nonetheless, transmission could still occur under the right conditions at the few racecourses that also host sport events, particularly if disinfection measures are inadequate. Multiple pathways are possible, including within‐population spread in the sport horse sector, highlighting the importance of standardising hygiene and biosecurity practices, especially at shared or high‐traffic facilities.

The occurrence of racing and sport events at separate venues within 24 h and 5 km of each other was relatively rare. The high RR values observed in several clusters indicate that the co‐occurrence of spatial proximity to a racecourse and temporal proximity to a race meeting was far more frequent within these clusters than would be expected if venues were randomly distributed in space and time. CIs would provide valuable insight into the precision of these RR estimates, but the traditional implementation of Kulldorff's spatial scan statistic does not generate CIs for RR. Recent work by Silva et al.^34^ describes a Monte Carlo method for determining these CIs, but as this is not yet available in standard software and requires substantial custom implementation and computational resources, it was not applied here. Future research could incorporate these methods to improve the statistical interpretability of cluster intensities. However, these elevated RRs reflect patterns of horse attendance at sport venues and racecourses over short timeframes, creating conditions that could facilitate pathogen spread between disciplines. Recognition of such spatiotemporal hotspots is important for informing targeted biosecurity measures and surveillance activities.

Previous studies have shown that equine influenza virus can travel up to 2 km in aerosol form, and that horses at premises within 5 km of the source may be at increased risk of infection.^14^, ^29^, ^35^ However, effective airborne transmission is contingent upon specific environmental conditions, including wind direction and speed, temperature, humidity, and hygiene practices. While racing and other equestrian disciplines predominantly function in discrete sectors, the presence of venues within 3 to 5 km of each other hosting events within a 24‐h window creates a theoretical possibility for cross‐sector pathogen transmission under conducive circumstances. Five kilometre spatial and 24‐h temporal thresholds were used in the current study as exploratory parameters to identify potential short‐term, localised clusters of venue‐events, with equine influenza serving as a proxy for contagious equine diseases due to its well‐characterised transmission dynamics. Our analysis therefore does not confirm any specific transmission pathway. Further investigation using mathematical modelling could identify optimal temporal thresholds for pathogens with varying transmission modes to quantify the risk of contagious equine disease spread within identified spatiotemporal clusters. In addition, without venue‐level data on horse movements, overnight stays or biosecurity practices, the clusters should be interpreted as potential short‐term, localised hotspots rather than proven epidemiological links.

This analysis provided a snapshot of competition attendance, rather than long‐term patterns. For example, a major southeast venue closed in 2019, and the COVID‐19 pandemic caused widespread disruption in venue use and event frequency. No data were available on direct contacts between horses attending events, or on horse movements between venues and home premises. Attendance at informal events, local rides, and cross‐border competitions was also not recorded. Similarly, the presence of non‐Thoroughbred horses at racecourses, such as police horses, hunter chasers, or participants in other breed races, was not captured, nor was the movement of pre‐trainers and breakers analysed. Human‐associated contact networks including grooms, transporters, and veterinary professionals were beyond the scope of this analysis. The recent growth of the equine industry in Great Britain may influence patterns of horse movement and participation across disciplines. Increased industry activity could modify opportunities for contact between horses, which in turn may affect the risk of pathogen transmission. Ongoing industry expansion underscores the need for continued monitoring and evaluation of cross‐discipline contact risks over time.

The absence of unique identifiers in the data from nonracing disciplines necessitated the assumption that horses with the same name across nonracing disciplines were the same individual. Although this may have led to occasional misclassification, this was nondifferential, and therefore unlikely to have introduced bias into the results. However, significant inconsistencies in horse identification within the showjumping dataset prevented us from tracking participation of showjumpers across disciplines. Given that showjumping venues are frequently used by multiple equestrian sports, this limitation will have contributed to an underestimation of inter‐discipline contact opportunities. Inconsistent horse identification systems and a fragmented approach to data collection across equestrian disciplines present significant obstacles to research, restricting our capacity to develop and implement a unified, data‐driven disease preparedness strategy, including contact tracing, for British horses. Widespread issues with equine passports further impede the accurate tracking of where horses reside, horse movements and owner contact details, underscoring the urgent need for standardised digital identification and record‐keeping systems. Achieving such standardisation, including consistent use of horse identifiers and comprehensive attendance records across all disciplines, is essential to enhancing the scope and precision of future epidemiological modelling and cross‐sector analyses. Continued collaboration among stakeholders and improvements in data capture practices will be vital for strengthening disease preparedness efforts across equine sectors. Future work should incorporate individual horse movement data to map contact networks and identify cross‐discipline participation. Other activities such as hacking out, or travelling to local shows, veterinary clinics, and riding schools, as well as the presence of resident horses at event venues, should also be considered, as they may contribute to the early spread of endemic or emerging diseases. Mapping the movement of people who work across sectors, including grooms, vets, farriers, and transporters, would help estimate human‐mediated risks, although all these would require considerable and sustained human engagement.

In conclusion, while racing and sport horse populations in Great Britain do largely operate separately, this study identifies infrequent instances of credible potential for local or indirect pathogen spread between racehorse and sport horse populations, particularly during peak racing and competition seasons, in areas with a high density of racecourses and equestrian competition venues.

## FUNDING INFORMATION

The study was funded by a small project grant from the Horserace Betting Levy Board (Ref: Sprj039). Tegan McGilvray's PhD studentship is funded by the Horse Trust (G3021).

## CONFLICT OF INTEREST STATEMENT

The authors declare no conflicts of interest.

## AUTHOR CONTRIBUTIONS

**Tegan A. McGilvray:** Conceptualization; investigation; writing – original draft; methodology; validation; writing – review and editing; software; formal analysis; project administration; data curation. **Kim B. Stevens:** Conceptualization; investigation; methodology; validation; writing – review and editing; software; formal analysis; supervision. **Kelsey L. Spence:** Conceptualization; investigation; methodology; writing – review and editing; supervision. **Sarah M. Rosanowski:** Conceptualization; writing – review and editing; supervision; investigation. **Josh Slater:** Conceptualization; writing – review and editing; supervision. **Jacqueline M. Cardwell:** Conceptualization; investigation; funding acquisition; methodology; validation; writing – review and editing; supervision; resources; project administration.

## DATA INTEGRITY STATEMENT

Tegan A. McGilvray had full access to all the data in the study and takes responsibility for the integrity of the data and the accuracy of the data analysis.

## ETHICAL ANIMAL RESEARCH

Ethical approval was obtained from the Royal Veterinary College Social Science Research Ethical Review Board: URN SR2025‐00872205.

## INFORMED CONSENT

Not applicable.

## Data Availability

The data that support the findings of this study may be available from the various sporting authorities involved in the study. Restrictions apply to the availability of these data, which were used under license for this study.
